# Practical Notes and Formulæ

**Published:** 1883-10-20

**Authors:** 


### PRACTICAL NOTES AND FORMULÆ.

**Eczema of the Face.**—In their work on the diagnosis and treatment of ocular affections, Messrs. Galezowski and Daguenet recommend, against the eczematous and impetiginous eruptions which often show themselves on the lids and nose of young patients suffering from phlyctenular keratitis, either calomel in powder, or the following ointment :

| | |
|---|---|
| Olei cadini | gr. iv, |
| Hydrarg oxid. rubr | gr. ij, |
| Camphoræ | gr. iv, |
| Vaselini | gr. clx. |

However, when there are many scabs, the best treatment consists in removing them with a forceps, and touching the denuded surface with a stick of nitrate of silver. The excess of caustic can be neutralized by the application of a solution of common salt.—*Louisville Med. News.*

**Acute Rheumatism.**—Dr. J. M. Granville advises no local applications except loose cotton-wool covered with light flannel ; no oil silk or other vapor proof material. He prescribes the following—

| | |
|---|---|
| ℞ Tr. aconiti (P. B.) | 12 minims, |
| Ammonia sulphide | 16 " |
| Aq. menth. virid. dist. | 6 ounces. |

M. Sig. One-fourth part every four hours, or in severe cases every three hours.

The sulphide of ammonia decomposes very easily, and therefore no more than four doses should be prescribed at one time.—*British Med. Jour.*

**Thompson's Eye Water.**—

| | |
|---|---|
| Sulphate of zinc | 20 grains, |
| Sulphate of copper | 5 grains, |
| Tincture of saffron | 2 drachms, |
| Tincture of camphor, | 1 drachm, |
| Rose water | 8 ounces, |
| Distilled water | 8 ounces. |

Mix and filter.

[It is difficult to understand the usefulness of such a mixture in any disease of the eye which could not be quite as well accomplished by a simple solution, of proper strength, of sulphate of zinc in distilled water.—*Ed. N. R.*]

**Squibb's Cholera Remedy.**—

| | |
|---|---|
| Chloroform | fl. ʒ 6, |
| Tincture of opium | fl. ℥ 2, |
| Spirit of camphor | fl. ℥ 2, |
| Tincture of capsicum | fl. ℥ 2, |
| Alcohol, sufficient to make | fl. ℥ 10. |

Dose, 40 to 60 drops, taken in water, each time bowels move loosely.

It is important in any case of diarrhœa, but is rarely appreciated except by physicians, that whenever there is too free action of the bowels, the patient should stop exercising and lie down—flat on the back—and stay there as quietly as possible. If there is pain, apply heat—dry heat is best.

---

In connection with the subject of cholera it has been said by Eli McClellan, Surgeon U. S. Army, one of the best authorities on the subject of cholera in this country, that the cholera poison requires an alkaline medium in which to develop. and that one of the best prophylactics is a mixture of aromatic sulphuric acid and laudanum ; two parts of the former to one part of the latter. For an adult, thirty drops ; for a child ten years old, ten drops ; and for a child under ten, five drops, in a little water every one, two, or three hours, as occasion may require.—*New Remedies.*

**Compound Parsley Mixture.**—This is a nervous sedative and tonic, recommended by Hammond. Its composition is as follows :

MISTURA APII COMPOSITA.

| | |
|---|---|
| ℞ Extract erythroxyli fl. | f ℥ 2, |
| Extract viburni (prun.) fl. | f ℥ 2, |
| Extract apii (graveol.) fl. | f ℥ 1. |

Misce. Dose, one to two teaspoonfuls three times a day.—*New Remedies.*

**Syrup of Coffee,** as used to disguise the bitter taste of quinine, can be made with coffee, roasted and finely ground, 4 oz.; alcohol, 1 oz.; sugar, 12 oz.; boiling water, sufficient. Pack the powder firmly into a percolator provided with a cover, and pour on boiling water until eight fluid ounces of percolate are obtained. Then dissolve the sugar [in the percolate] by percolation, and finally add the alcohol as a preservative. The taste of two grains of quinine is said to be pretty well covered by a drachm of the syrup.—*Ibid.*

**Creasote Wine.**—

| | | |
|---|---|---|
| Wood creasote | 6 | parts, |
| Compound tinct. of gentian | 30 | " |
| Alcohol | 230 | " |
| Sherry wine | 710 | " |

Mix. Dose, a tablespoonful two or three times daily in a cupful of water.

Recommended in phthisis, to diminish cough, fever, expectoration, etc.—*Ibid.*

**S. S. S.**—This popular nostrum, which has been advertised *ad nauseam*, is said to be composed of the following ingredients—originally prepared by an old Indian Chief, Billy Bowlegs, of Florida: Chionanthus Virg., a bushel of the root; Xanthoxylum frax., one pound; Rhus glabra (and black sumach), R. typhirea, *aa.* one-half pound; Sarsaparilla root, ten ounces. All boiled strong in eight gallons of water, then an ounce of cupri sulph. added, and given in doses of one ounce three times a day.

**To Allay the Itching in Eczema.**—A good local application, which is both protective and stimulant, is to cover the surface of the body with an ointment made as follows:

℞ Zinci oxidi.................................ʒ j

Olei juniperi.................................ʒ j

Adipis.................................℥ i

M. Sig. Use as an ointment.

**Remedy for Earache.**—In the Druggists' Circular for July, 1883, a correspondent says:

The remedy which I here offer has, after repeated trials, never failed to afford almost instant relief. It is perfectly simple, easy of application, costs but little, and can be procured at any drug store.

Here it is with accompanying directions:

℞ Olive oil.................................ʒ j

Chloroform.................................℥ j

Mix and shake well together; then pour twenty-five or thirty drops into the ear and close it up with a piece of raw cotton to exclude the air and retain the mixture.

The remedy I can truly say is a specific in earache. It acts promptly and efficiently, and in my hands has never failed to effect a cure in a short space of time.—*Med. and Surg. Rep.*

**Hydrated Oxide of Iron.**—Dr. Squibb recommends the following as a simple method of preparing Hydrated Oxide of Iron, the antidote for arsenic, one of its chief advantages being that the ingredients are always easily obtained. Take of

Tinct. Ferri Chloridi...................℥ iv,

Aquæ Font...................℥ iv,

Mix in a vessel of...................℥ xij capacity,

And add aqua ammon...................℥ ij.

Shake well, pour it on a large wet muslin drainer, wring out the water and alcohol and wash with fresh water. The stomach having been evacuated by emetics while the antidote was being prepared, give f. ʒ iv at once, to be followed by an emetic. Then give ʒ ij every ten minutes.—*Pacific Med. and Surg. Journal.*

**Whooping Cough Specific.**—℞. Acid nitrici dil, ʒ j; tr. cardamon co. ʒ iij; syrup simplicis ʒ iv; aque ad, ℥ iv. M. Take a dessert spoonful every four hours.—*Amer. Med. Jour.*

**Flatulent Dyspepsia.**—

R. Potass. chlor............................2½ drachms,

Sodæ bicarb..............................2½ drachms,

Rhei pulv.................................½ drachm,

Capsici pulv..............................4 grains,

Ol. sassafras.............................2 drops.

M. Sig. Dissolve in half pint of water, and give tablespoonful immediately after each meal.—*Med. Brief, Dec.*

**Intense Itching.**—Sponge the parts once or twice a day with pure rectified spirits, containing five minims of carbolic acid to the ounce.—*Dr. James Startin, in the Lancet.*

**Sir Benjamin Brodie's Prescription for Gout.**—

℞ Pil. hydrargyri, }

Ext. rhei, } ..............................aa ℈ j,

Ext. coloc. co., }

Ext. colchici acet..............................grs. xv.

Ft. pil. xv. Sumantur tres horæ somni pro renata—*Louisville Medical News.*

**Tetter of the Hands.**—Dr. Crum (in Brief) says: "I would advise the use of either of the following ointments:

'℞ Chrysophanic acid..............................ʒ j,

Cosmoline..............................℥ j.

M. Sig. Apply to affected parts night and morning.'

'℞ Oxalic acid..............................ʒ j,

Pure glycerine..............................℥ iij.

M. Sig. Apply night and morning.'"

**Resorcin in Cholera Infantum.**—In Breslau, ninety-one cases of cholera infantum were treated with resorcin in the dose of one-third to one-half grain in two ounces of infusion of chamomile. The success of this treatment was remarkable. How often the dose was given is not stated.—*Med. Times, Dec. 17.*

**To Prevent Pitting in Smallpox.**—A writer (in Southern Clinic) recommends, to prevent pitting in smallpox, the following:

Take of white lead (plumbum carbonicum), quantum lib., mix with linseed oil q s. to make a cream-like paste, add to the bulk about five to six per cent. carbolic acid, and apply with a large camel's-hair brush repeatedly, so as to keep the surface of the face, hands, etc., permanently and fully covered.

**Urticaria.**—Dr. Ashworth (in Medical Brief) recommends:

"℞ Pure absolute alcohol..............................½ drachm,

Ol. tiglii..............................1 drop.

M. Sig. Five to ten drops every three or four hours."

